# Quantification of damage due to low-dose radiation exposure in mice: construction and application of a biodosimetric model using mRNA indicators in circulating white blood cells

**DOI:** 10.1093/jrr/rrv066

**Published:** 2015-11-19

**Authors:** Hiroshi Ishihara, Izumi Tanaka, Haruko Yakumaru, Mika Tanaka, Kazuko Yokochi, Kumiko Fukutsu, Katsushi Tajima, Mayumi Nishimura, Yoshiya Shimada, Makoto Akashi

**Affiliations:** 1 Research Center for Radiation Emergency Medicine, National Institute of Radiological Sciences, 4-9-1 Anagawa, Inage-ku, Chiba 263-8555, Japan; 2 Research Center for Radiation Protection, National Institute of Radiological Sciences, 4-9-1 Anagawa, Inage-ku, Chiba 263-8555, Japan; 3 Board, National Institute of Radiological Sciences, 4-9-1 Anagawa, Inage-ku, Chiba 263-8555, Japan

**Keywords:** circadian rhythm, *Cdkn1a*, *Bbc3*, *Bax*, *Myc*, cysteamine

## Abstract

Biodosimetry, the measurement of radiation damage in a biologic sample, is a reliable tool for increasing the accuracy of dose estimation. Although established chromosome analyses are suitable for estimating the absorbed dose after high-dose irradiation, biodosimetric methodology to measure damage following low-dose exposure is underdeveloped. RNA analysis of circulating blood containing radiation-sensitive cells is a candidate biodosimetry method. Here we quantified RNA from a small amount of blood isolated from mice following low-dose body irradiation (<0.5 Gy) aimed at developing biodosimetric tools for situations that are difficult to study in humans. By focusing on radiation-sensitive undifferentiated cells in the blood based on *Myc* RNA expression, we quantified the relative levels of RNA for DNA damage-induced (DDI) genes, such as *Bax* , *Bbc3* and *Cdkn1a* . The RNA ratios of DDI genes/ *Myc* in the blood increased in a dose-dependent manner 4 h after whole-body irradiation at doses ranging from 0.1 to 0.5 Gy (air-kerma) of X-rays, regardless of whether the mice were in an active or resting state. The RNA ratios were significantly increased after 0.014 Gy (air-kerma) of single X-ray irradiation. The RNA ratios were directly proportional to the absorbed doses in water ranging from 0.1 to 0.5 Gy, based on gamma-irradiation from ^137^ Cs. Four hours after continuous irradiation with gamma-rays or by internal contamination with a beta-emitter, the increased RNA ratios resembled those following single irradiation. These findings indicate that the RNA status can be utilized as a biodosimetric tool to estimate low-dose radiation when focusing on undifferentiated cells in blood.

## INTRODUCTION

Serious accidents at massive nuclear power plants lead to low-dose irradiation of a large number of victims. Equivalent doses to the victims due to the accidents can be estimated physically using the radiation dose rate of the evacuation route. For more precise dose estimation, however, biodosimetric data using biologic samples isolated from the victims are desirable. Dicentric chromosome analysis of lymphocytes is the most practicable method [ 1 ] and is useful for estimating radiation dose after exposure to high-dose radiation [ 2 , 3 ]. This method is not easily applied for low doses of around or below 100 mGy, however, although the method was applied to workers in the Tokyo Electric Power Company Fukushima-1 nuclear power plant in 2011 [ 4 ]. Before measuring chromosomal damage by low-dose irradiation following an accident, lymphoblasts may have already accumulated damage due to previous medical exposure and natural radiation [ 5 ]. Therefore, it is necessary to develop and establish an alternative biodosimetric methodology to supplement chromosomal analysis, focusing on low-dose radiation.

Measurement of γH2ax foci in chromosomes can be used to determine radiation-induced damage from a wide-range of doses [ 6 ]. Practical application of the method for biodosimetry is not easy, however, because measurable foci disappear within a few hours of exposure. As an alternative method of measuring radiation damage, RNA analysis of blood cells has been studied [ 7 ]. Various RNA species are listed as damage-indicators by microarray analyses using *ex vivo* irradiated human blood [ 8–14 ] and blood isolated from patients after medical exposure [ 15 , 16 ]. RNA quantification is a candidate biodosimetric method for detecting exposure to low doses (∼20 mGy) [ 13 , 17 ], but the RNA status quickly changes after irradiation. RNA studies are also used to reveal damage-response mechanisms because alternative splicing [ 18 , 19 ] and non-protein-coding RNAs, such as miRNA [ 20 ], are also induced after irradiation.

Experimental animal models can be used to study various modes of body-radiation that are difficult to evaluate in humans. The experimental data using animals of various ages and inbred strains may give us clues to speculate differences in biodosimetric parameters by the age and race in human beings. In mice [ 21 , 22 ], ratios of mRNAs for DNA-damage-induced (DDI) genes such as *Cdkn1a* ( *P21* , *Waf* ), *Mdm2* , *Bax* and *Bbc3* ( *PUMA* ) per housekeeping gene, such as glyceraldehyde-3-phosphate dehydrogenase gene ( *Gapdh* ), are increased in blood cells in a dose-dependent manner. The radiation-dose dependency of these mRNAs ratios, however, varies ∼2-fold between daytime and nighttime [ 22 ], suggesting that RNA ratios in the blood may differ between active and resting humans. In the present study, we reduced the effect of circadian rhythm on measurements of radiation-induced RNA expression of DDI genes in mice, and demonstrated that the DDI gene/ *Myc* RNA ratios linearly increased following low-dose radiation from 0.1 to 0.5 Gy in undifferentiated cells in the circulating blood both during the day and night. Using the biodosimetric model, we compared the DDI gene/ *Myc* RNA ratios after both a single exposure and continuous exposure. Effects on the RNA status of the radioprotector aminothiol were also examined.

## MATERIALS AND METHODS

### Mouse treatment

Mice were treated in accordance with the Guidelines for the Proper Conduct of Animal Experiments (Science Council of Japan), and the Institutional Animal Care and Use Committee approved the study. Male C3H/He inbred mice at 7 weeks of age (Japan SLC Inc., Shizuoka, Japan) were acclimatized at 3–5 mice per cage for 2 weeks in an animal room maintained at 23°C and 55% humidity. Mice were housed on a 12 h:12 h light:dark schedule, with white light on at 10–50 lux during the day. In the nighttime handling of mice, procedures were performed under blue LED light of <0.4 lux. The radioprotector cysteamine HCl (Wako Pure Chem. Co., 3 mmole/kg) and the anesthetic pentobarbital sodium salt (Nacalai Tesque Co., 76 mg/kg) were injected subcutaneously.

### Mouse exposure to external and internal radiation

Fewer than 10 mice were simultaneously whole-body irradiated using a cylindrical irradiation container [ 22 , 23 ]. For nighttime irradiation, the container was covered with a blackout bag. For abdominal irradiation, anesthetized mice shielded from chest to head were irradiated [ 24 , 25 ]. An ionization chamber was placed in a concentric irradiation field more than 5 cm away from the mice to avoid counting scattered radiation from the mouse body. The mice were irradiated using an X-ray generator (PANTAK HF-320, Shimadzu Corp., Japan) at 200 V, 20 A and filtered at 0.5 cm Al/Cu, while measuring with the ionization chamber at a focus-sample distance of 1000 mm (only for irradiation ≤100 mGy) or 700 mm with a dose rate of kinetic energy released per unit mass (air-kerma) of 0.25 or 0.40 Gy/min, respectively. The radiation dose rate of the irradiation field was inspected and controlled by the Department of Technical Support and Development of our Institute

For single exposure to gamma-rays from ^137^ Cs, the mice were placed in a cylindrical irradiation container and irradiated in a field with an estimated absorbed dose to water (EDw) rate of 0.535 Gy/min. For continuous irradiation, mice reared in cages were irradiated in a field of 0.032 mGy/h (EDw) of ^137^ Cs gamma-rays. These EDw values were determined by ionizing chamber measurement using a phantom mouse and the air-kerma values of the field, as measured by the Department of Technical Support and Development at our institute. For internal contamination experiments, ^32^ P-NaH _2_ PO _4_ (MP Biochemicals, Japan Radioisotope Association, 3.7 TBq/mmole) in phosphate-buffered saline solution was subcutaneously injected at doses of 80 or 200 MBq/kg-body weight.

### Dose estimation by ^32^ P-phosphate internal exposure

Based on the assumption that ^32^ P was homogenously dispersed and all the energy from the β-rays was absorbed in the mouse, the EDw was estimated using the formula:

Eq:1 D(t)=E×C0×tηfor tritium,

where C0 = initial concentration (Bq/kg) and *η* = modified factor [ 26 ]. The mean energy (E) of ^32^ P is 695 keV (1.11 × 10 ^−13^ J). When the coefficient of *η* was 1.00, injections of 200 MBq/kg or 80 MBq/kg provided an EDw of 0.080 or 0.032 Gy/h, respectively.

### RNA preparation and quantification

The details for accurate quantification of blood RNA were described previously [ 22 ]. In brief, total RNA was prepared using an RNeasy Mini Kit® with DNase I (Qiagen) from 0.05 ml of circulating blood collected from the murine tail in a microtube containing EDTA (final concentration: 10 mM). The cDNA synthesis reaction contained total RNA (300 ng), AMV reverse transcriptase (10 units, Takara Bio Inc., Japan), and RS19-dT15 primer (1.25 pmol, Table 1 ) [ 26 ]. Quantitative measurement of real-time PCR (Q-PCR) was performed using SYBR® Green PCR Master Mix and ABI Prism7500Fast® Sequence Detection System (Applied Biosystems Inc., Foster City, CA, USA) as described previously [ 22 ]. All the cDNA samples were amplified in quadruplicate. As template cDNA, the initial amounts of total RNA per well for the quantification of *Bax* , *Bbc3* , *Cdkn1a* and *Myc* were 2–8 ng, 5–20 ng, 5–50 ng and 10–100 ng, respectively. As a reference, linear plasmid DNA corresponding to similar molar amounts of all the Q-PCR target nucleotide sequences at 10 zeptomoles per well were used in all the reactions. Table 1 lists the primers used for the Q-PCR and for the preparation of reference DNA. Relative mean value of the converted threshold cycle was used, as reported previously [ 22 , 25 , 27 ].

**Table 1.** DNA Primers used in this study

Primer name Sequence
*Universal for cDNA synthesis, RS19-dT15*
5′-AGCACTGACCGATGTCACG-(T)15-3′
*Primers for Q-PCR*
*Bax-Fw* 5′-TGCTAGCAAACTGGTGCTCAA-3′
*Bax-Rv* 5′-AGCCCATGATGGTTCTGATCAGCT-3′
*Bbc3-Fw* 5′-ACCTCAACGCGCAGTACGA-3′
*Bbc3-Rv* 5′-TGAGGGTCGGTGTCGATGCT-3′
*Cdkn1a-Fw* 5′-GAGGCAGACCAGCCTGACA-3′
*Cdkn1a-Rv* 5′-TCTGCGCTTGGAGTGATAGAAAT-3′
*Myc-Fw* 5′-AGCCCCTAGTGCTGCATGA-3′
*Myc-Rv* 5′-GTTTGCCTCTTCTCCACAGACAC-3′
*Gapdh-Fw* 5′-TGGCCAAGGTCATCCATGACAAC-3′
*Gapdh-Rv* 5′-TCCAGAGGGGCCATCCACAGTCTTCTG-3′
*Pfx(high-fidelity)-PCR primers for reference preparation*
*Bax-Fw* 5′-TGCTGACGTGGACACGGA-3′
*Bax-Rv* 5′-ACAAAGATGGTCACTGTCTGCCATG-3′
*Bbc3-Fw* 5′-ACCACAACGCGCAGTACGA-3′
*Bbc3-Rv* 5′-AGTCCAGTATGCTACATGGTGCA-3′
*Cdkn1a-Fw* 5′-AGGCCCGAGAACGGTGGAACTTT-3′
*Cdkn1a-Rv* 5′-TCTGCGCTTGGAGTGATAGAAAT-3′
*Myc-Fw* 5′-AATCCTGTACCTCGTCCGATTC-3′
*Myc-Rv* 5′-ACCCTGCCACTGTCCAACT-3′
*Gapdh-Fw* 5′-GTCTTCACCACCATGGAGAAG-3′
*Gapdh-Rv* 5′-TCCAGAGGGGCCATCCACAGTCTTCTG-3′

### Statistics

Significant differences were determined by Dunnett's test following one-way analysis of variance. For regression analyses to determine the correlation coefficient, regression line and confidence interval, macros (StatMateIII, ATMS Co. Tokyo) of Microsoft® Excel® software were used.

## RESULTS

### Variation of indicator values in circulating white blood cells based on mouse activity

**Fig. 1.**
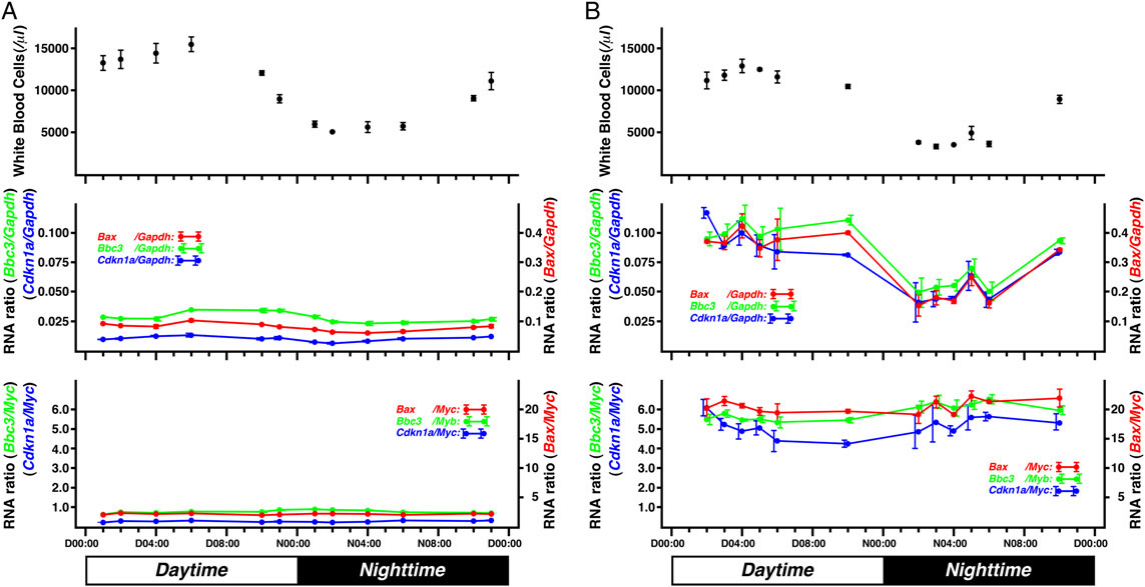
Circadian rhythm of indicators in circulating blood. ( **A** ) Indicators in blood collected from mouse tails 4 h after sham irradiation. The horizontal axis corresponds to the blood collection times. Concentration of WBC per microliter (upper panel), and the *Bax* / *Gapdh* (red dots and line, middle panel), *Bbc3* / *Gapdh* (green dots and line in middle) and *Cdkn1a* / *Gapdh* (blue dots and line in middle) RNA ratios are indicated. In the lower panel, *Bax* / *Myc* (red dots and line), *Bbc3* / *Myc* (green dots and line) and *Cdkn1a* / *Myc* (blue dots and line) RNA ratios are indicated. ( **B** ) Effect of circadian rhythm on the indicators in circulating blood collected from mouse tails 4 h after whole-body X-irradiation at a dose of 0.5 Gy. Concentrations of WBC (upper panel), DDI gene/ *Gapdh* (middle), and DDI genes/ *Myc* (lower) RNA ratios are shown. Means and standard errors of three to four mice per time-point are indicated.

The need to collect only a small amount of peripheral blood would be advantageous following an enormous radiation disaster. The levels of intracellular molecules that reflect radiation damage in the specimen vary during storage. When the specimen is treated immediately after collection, however, the levels of the intracellular molecules can be maintained in storage for a long time. For practical future application of the mouse model, we focused on RNA of mice exposed to low-dose irradiation using a small volume of peripheral blood from the tail. When using circulating blood as a sample, the concentration of white blood cells (WBCs) drastically changed during the day in accordance with the circadian rhythm (Fig. 1 A, upper). The mean WBC concentration in resting mice during the day was 14 136 ± 1673 cells/μl, and in active mice at night it was 5576 ± 668 cells/μl, though the concentration of red blood cells (RBCs; 9703 ± 371 cells/nl) did not change (data not shown). Although the WBC concentration decreased at 4 h after 0.5 Gy X-irradiation (daytime mean of 12 043 ± 1236 and nighttime mean of 3796 ± 792 cells/μl), the large difference in the WBC concentration between daytime and nighttime was maintained (Fig. 1 B, upper). This suggests that the concentration of radiation-sensitive cells in the WBCs also fluctuated between daytime and nighttime.

**Fig. 2.**
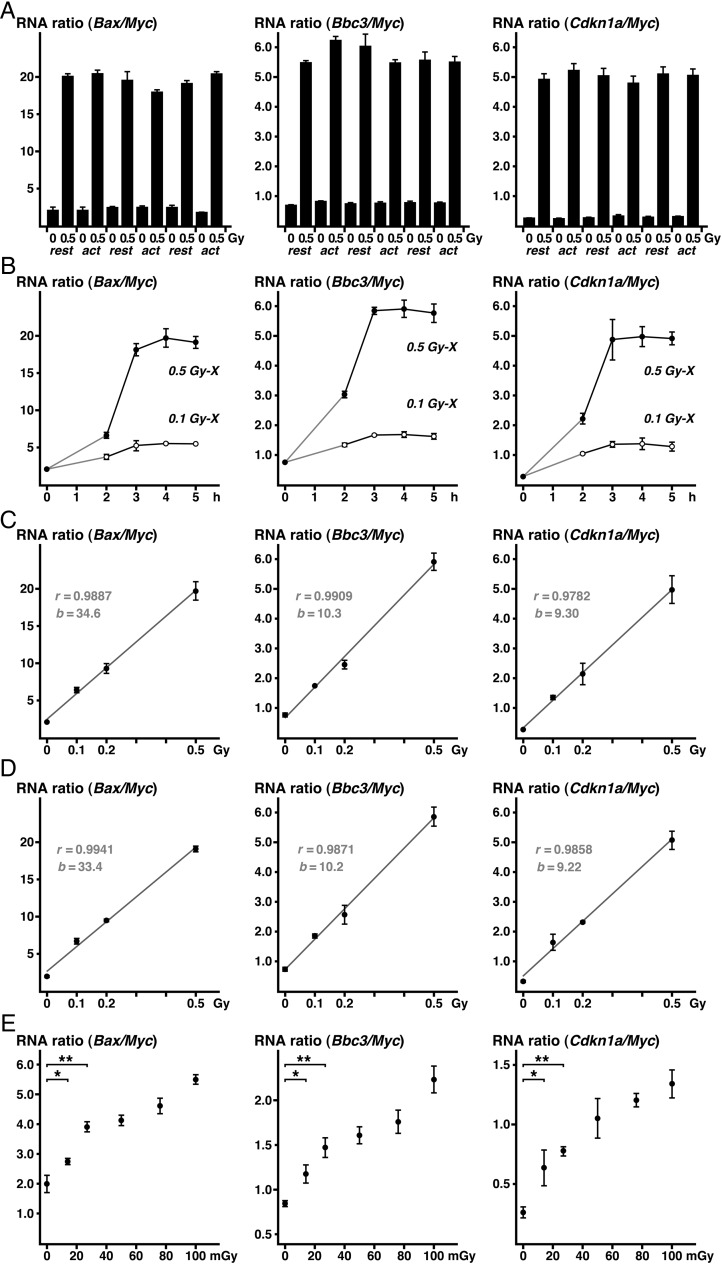
The DDI gene/ *Myc* RNA ratios in WBC collected 4 h after X-irradiation. ( **A** ) Comparison of the *Bax* / *Myc* (left), *Bbc3* / *Myc* (middle) and *Cdkn1a* / *Myc* (right) RNA ratios in blood collected 4 h after sham (0) or 0.5 Gy (air-kerma) of X-irradiation (0.5) of mice in a resting (rest) or active (act) state among three different production batches (Batch-1 to 3). ( **B** ) Time-course of the DDI gene/ *Myc* RNA ratios after X-irradiation at the air-kerma of 0.5 Gy (closed circles) or 0.1 Gy (open circles). ( **C** ) Correlation between the DDI gene/ *Myc* (vertical axis) RNA ratios and exposed doses of X-ray (air-kerma, horizontal axis) in WBC collected from active mice. Regression lines, correlation coefficients ( *r* ), and slopes of the lines ( *b* ) are indicated in gray color. ( **D** ) Correlation between DDI gene/ *Myc* RNA ratios and exposure doses of X-rays in WBC from resting mice. Regression lines, correlation coefficients ( *r* ), and slopes of the lines ( *b* ) are shown. ( **E** ) Correlation between the DDI gene/ *Myc* RNA ratios and exposure doses of low-dose *X-* rays. Significance compared with sham irradiation *( *P* < 0.05) or **( *P* < 0.01). Means and standard errors are shown.

The mRNA levels of the DDI genes *Cdkn1a* , *Bax* and *Bbc3* are reported to be indicators of radiation-induced damage [ 20 , 21 , 28–30 ]. *Cdkn1a* mRNA is a growth-arrest indicator, and *Bax* and *Bbc3* mRNAs are expressed during early apoptosis. These DDI genes are expressed in radiation-sensitive undifferentiated cells, which are presumed to be scarce in circulating WBCs ( Supplementary Data ). On the other hand, mRNA for housekeeping genes such as *Gapdh* , which is used frequently as a reference gene, is expressed in all living WBCs. Therefore, the ratio of DDI gene RNA to *Gapdh* RNA is influenced by the concentration of radiation-sensitive cells in WBCs. In fact, circadian changes were also observed in the DDI gene/ *Gapdh* RNA ratio (Fig. 1 B, middle), as reported earlier [ 22 ]. To avoid the circadian change in the rate of undifferentiated cells in circulating WBCs, we focused on the mRNA for the *c-myc* proto-oncogene ( *Myc* ), which is expressed predominantly in undifferentiated cells ( Supplementary Data ) [ 31 , 32 ], which are thought to be radiation-sensitive. Therefore, the DDI gene/ *Myc* RNA ratio in total WBCs is expected to reflect the damage level of radiation-sensitive cells. In fact, the RNA ratios were not influenced by circadian cycle without (Fig. 1 A, lower) or after irradiation (Fig. 1 B, lower) among the batches of mice (Fig. 2 A). Thus, we used these RNA ratios as damage indicators after body irradiation in mice.

### Dose-dependent increase in DDI gene/ *Myc* RNA ratios

The DDI gene/ *Myc* RNA ratios in WBCs from mice after whole-body X-irradiation of 0.1 or 0.5 Gy (air-kerma) are shown in Fig. 2 B. The ratios increased and peaked 3 h after irradiation, and the peak level was maintained for at least 5 h after irradiation. The peak levels decreased at 8 h and gradually declined until 24 h ( Supplementary Data ). The ratios returned to the background level 7 days after irradiation at 0.5 Gy. Because the variation between individuals in the data for blood collected during the time of the decrease was larger than that for blood collected at the peak time, we collected the blood at 4 h after irradiation in the middle of the peaks of the RNA ratios. Figure 2 C and D show the relationship between the X-ray dose and RNA ratios at the middle of the peak at 4 h after irradiation in mice during the daytime (Fig. 2 C) and nighttime (Fig. 2 D). Results of the regression analysis indicate that the regression coefficients during the daytime (Fig. 2 C) and nighttime (Fig. 2 D) were closely related, demonstrating that radiation damage in WBCs can be estimated by measuring the DDI gene/ *Myc* RNA ratios, regardless of whether mice are in an active or resting state. Mice exposed to low doses of X-rays (14–100 mGy, air-kerma) had significantly higher RNA ratios in their blood (Fig. 2 E).

### Effect of radioprotector and abdominal irradiation

**Fig. 3.**
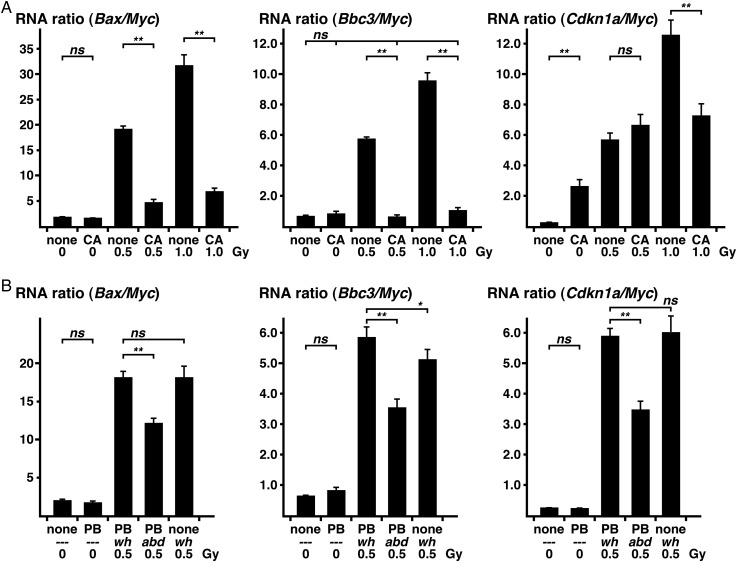
Effect of radioprotector or abdominal exposure on the DDI gene/ *Myc* RNA ratios in WBC. ( **A** ) Mice were irradiated at the indicated air-kerma dose of X-ray, 30 min after injection of cysteamine (CA) or saline (none). Four hours after irradiation, blood was collected for RNA quantification. ( **B** ) Anesthetized (PB) or non-anesthetized (none) mice were sham (0) or X-irradiated (0.5) at the air-kerma dose of 0.5 Gy. Four hours after whole-body irradiation (wh) or abdominal irradiation (abd), blood samples were collected. Significance of data compared with sham-irradiation are shown as ns (not significant), *(P < 0.05), or **(P < 0.01). Means and standard errors are shown.

The RNA ratios in circulating WBCs were used to study the effects of a radioprotector *in vivo* . Aminothiol is a radioprotector that neutralizes the toxicity of reactive oxygen species generated by radiation exposure. We injected aminothiol cysteamine into mice 30 min before irradiation, and blood was collected 4 h after exposure. The increases in the *Bax* / *Myc* and *Bbc3* / *Myc* RNA ratios induced by X-irradiation were drastically reduced by pre-exposure injection of cysteamine (Fig. 3 A). The *Cdkn1a* / *Myc* RNA ratio, however, was augmented by cysteamine injection, and cysteamine did not prevent the increase in the RNA ratio induced by 0.5 Gy irradiation. Inhibition of the increase in the RNA ratio by cysteamine was observed only when the mice were exposed to a higher dose (1.0 Gy air-kerma) of X-rays.

The DDI RNA ratios in circulating WBCs are expected to be a useful dosimetry tool for localized irradiation, because WBCs exposed in local tissue are presumed to be dispersed throughout the circulatory system before blood collection. To confirm this, approximately two-thirds of the mouse body was irradiated during abdominal irradiation in mice anesthetized with pentobarbital, which does not affect DDI gene expression (Figs. 2 B, 3 B). All of the RNA ratios in the WBCs at 4 h after abdominal irradiation were decreased compared with that after whole-body irradiation (Fig. 3 B).

### Comparison of X-ray and gamma-ray irradiation

**Fig. 4.**
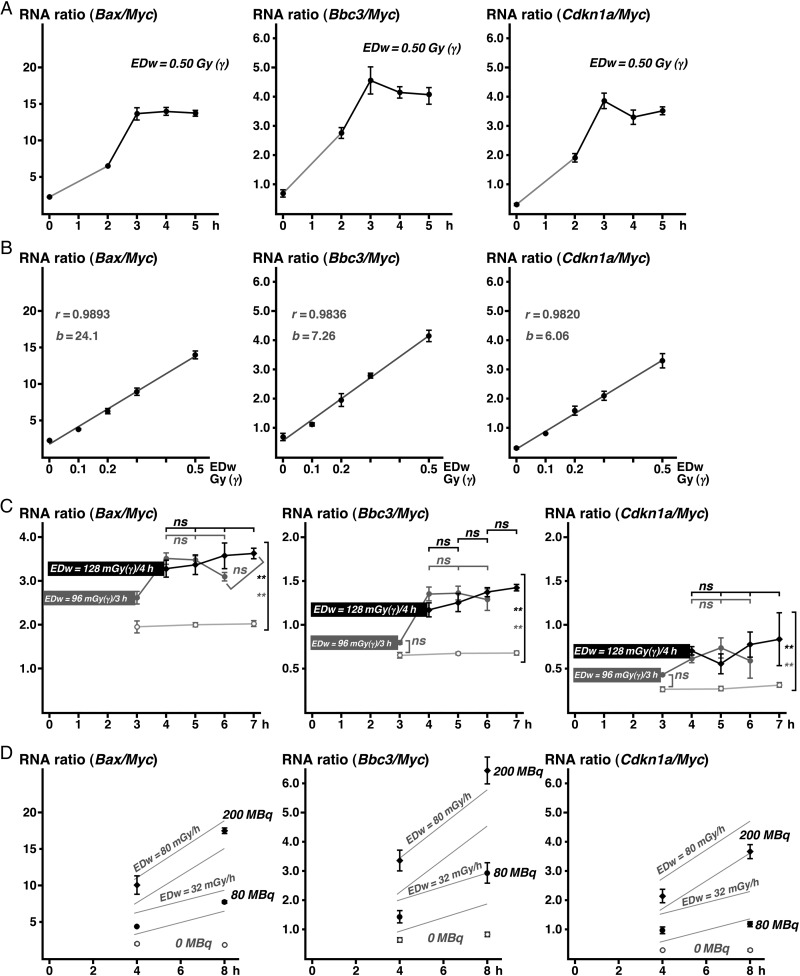
Examples of the DDI gene/ *Myc* RNA ratios in WBC after single γ-irradiation, continuous γ-irradiation, and continuous β-irradiation. ( **A** ) Time-course of the DDI gene/ *Myc* RNA ratios after γ-irradiation at an EDw of 0.5 Gy. ( **B** ) Correlation between the DDI gene/ *Myc* RNA ratios (vertical axis) and γ-ray exposure doses (EDw, horizontal axis) in WBC collected from mice 4 h after a single exposure. Regression lines, correlation coefficients (r), and slopes of the lines (b) are indicated in gray color. ( **C** ) Time-course of the DDI RNA ratios following continuous γ-irradiation for 3 h at a total EDw of 96 mGy (dark-gray box, circles, and line), for 4 h at total EDw of 128 mGy (black box, circles, and line), or for 4 h with sham-irradiation (light gray circles and line). Horizontal axis indicates times of irradiation (from 0 - 3, or from 0 - 4) and of blood collection. ( **D** ) Time-course of the RNA ratios following continuous internal exposure of β-rays with injected ^32^ P at a dose of 200 M (black diamonds), 80 M (black closed circle), or 0 Bq/kg (gray open circle). Gray line indicates 95% confidence intervals of the predicted RNA ratio corresponding the EDw-rates, calculated from the data obtained from the single γ-irradiation, as shown in Fig. 2 B.

The relation between the absorbed dose in WBCs and the exposed dose (air-kerma) varies following whole-body irradiation among different X-ray generators, because the radiation quality (i.e. a combination of various wavelengths of electromagnetic waves) from X-rays varies among generators. Based on the assumption that DNA damage in WBCs correlates with the EDw of radiation, mice were irradiated with a gamma-ray irradiator of ^137^ Cs in which the EDw was controlled. The profiles of these RNA ratios in collected blood following whole-body irradiation with gamma-rays (Fig. 4 A) were similar to those following whole-body irradiation with X-rays (Fig. 2 B). The correlations between the DDI RNA ratios and EDw from 0.1 to 0.5 Gy are shown in Fig. 4 B.

### Effect of continuous irradiation

There was an interval between the increase in biologic indicators, such as the DDI RNA ratios, and irradiation. After a single irradiation for <2 min, the RNA ratios peaked at 3 h and were maintained for at least 5 h after exposure (Figs 2 B and 4 A). Continuous exposure at a low-dose rate is thought to further delay the time-dependent responses of the RNA ratios. To examine whether exposure of 0.1 Gy at a low dose rate is detectable based on the DDI RNA ratios, we exposed the mice in a γ-irradiation field at an EDw of 0.032 Gy/h for 3 h (total EDw of 0.096 Gy) or 4 h (EDw = 0.128 Gy). The DDI RNA ratios in the WBCs of these mice were significantly increased 4 h after the initial exposure, compared with RNA ratios in sham-irradiated mice (Fig. 4 C). The peak levels of these RNA ratios (Fig. 4 C) were below the peak levels expected from the regression line obtained after a single irradiation (Fig. 4 B). The difference in the RNA ratios was not significant between 0.096 Gy- and 0.128 Gy-irradiated mice (Fig. 4 C).

A continuous irradiation model was further used to study the internal contamination with ^32^ P. Injection of ^32^ P-PO _4_ at 200 MBq/kg or 80 MBq/kg gave an EDw of 0.080 Gy/h or 0.032 Gy/h, respectively, when the ^32^ P radionuclide was homogenously dispersed, as described in the Materials and Methods. We observed dose-dependent increases in all of the DDI RNA ratios in WBCs from the injected mice (Fig. 4 D).

## DISCUSSION

We demonstrated that the *Bax/Myc* , *Bbc3/Myc* or *Cdkn1a* / *Myc* RNA ratios in WBCs can be used for quantitative determination of damage due to low-dose radiation in mice. Experimental animals exposed to body irradiation can provide various biologic data that are difficult to evaluate from human samples. We focused on low-dose exposure for two reasons. The first reason, is that biodosimetry following low-dose exposure is not easy to perform using the previously established chromosomal analysis [ 1–5 ]. If biodosimetric techniques for low-dose exposure could be established, the method could be utilized to monitor workers in various industries. The second reason is that the biologic mechanisms after low-dose radiation exposure are assumed to be distinct from those following high-dose exposure, which induces a number of cell death events leading to various signal transduction pathways.

The use of RNA status for biodosimetry is advantageous because the data can be obtained rapidly using only a small number of cells, as compared with chromosomal analysis. The choice of indicator RNA is essential for establishing the methodology to measure cellular damage. In this study, we used mRNAs of the well-known genes *Bax* , *Bbc3* and *Cdkn1a* , which are considered biodosimetric indicators following low-dose irradiation in human [ 7 , 8 , 10 , 12 , 16 , 17 , 33 , 34 ] and mouse [ 20 ] cells. DNA damage by radiation in proliferative cells activates p53 protein via ATM signals, and then the p53 activates the growth-arrest protein CDKN1A and proapoptotic proteins BAX and BBC3 [ 35 ]. Simultaneously, the activated p53 protein transcriptionally induces various genes, including *Cdkn1a* , *Bax* and *Bbc3* [ 36 ]. Therefore, these RNAs can be used as damage indicators reflecting biologic processes leading to apoptosis.

### Minimization of the differences in the RNA status between daytime and nighttime

The above-mentioned three DDI genes are expressed in undifferentiated cells after irradiation, and cause growth arrest or cell apoptosis. We used *Myc* mRNA as an indicator of the number of undifferentiated WBCs [ 23 , 24 ] to reduce the effect of differentiated cells in WBCs on the RNA status. Circadian fluctuations in the DDI gene RNA ratios relative to the *Gapdh* after irradiation (Fig. 1 B middle) [ 22 ] were minimized by using the DDI gene/ *Myc* RNA ratios, regardless of whether the mice were in an active state or resting state (Fig. 1 B bottom, Fig. 2 A). If there are differences in the concentrations of undifferentiated cells in collected human blood between resting patients and physically active persons (e.g. first responders), it is necessary to use RNAs for DDI genes related to that of a suitable undifferentiated cell indicator for biodosimetric quantification, not a house-keeping gene or total RNA.

### Studies using single irradiation models

The RNA ratios of DDI genes/ *Myc* peaked 3 h after single irradiation with X-rays and gamma-rays (Figs 2 B and 4 A). The peak RNA ratios after abdominal irradiation were approximately half that after whole-body irradiation (Fig. 3 B), suggesting that the RNA ratios can be used to estimate WBC damage after localized irradiation. The RNA ratios quantitatively increased after a single X-irradiation at a dose of 0.1 to 0.5 Gy (air-kerma) for both daytime and nighttime exposure (Fig. 2 C and D). Based on the correlation between the RNA ratios and EDw (Fig. 4 B), the coefficients of EDw/air-kerma of X-rays calculated using the RNA ratios of *Bax* / *Myc* , *Bbc3* / *Myc* and *Cdkn1a* / *Myc* were 1.29, 1.28 and 1.37, respectively. It is presumed that the larger coefficient in circulating blood is generated by the scattered radiation of X-rays.

Mean double-stranded break (DSB) numbers in a single cell per 1 Gy are estimated to be ∼21 [ 37 ] or ∼35 [ 38 ] by γH2Ax foci analyses. Because it is estimated that ∼40 DSBs are necessary for lethal damage to a cell [ 39 ], cell death in WBCs is not expected to be readily visible following irradiation in the range from 0.1 to 0.5 Gy. The linearity suggests that the RNA ratios are suitable indicators of the invisible damage in WBCs exposed to the range from 0.1 to 0.5 Gy.

The linearity of the DDI RNA ratios and exposed dose declined after low-dose radiation below 100 mGy (Fig. 2 E). Because such low-level exposure is estimated to be below the lethal dose for WBCs, undetermined biologic responses may affect the expression levels of these DDI genes. Significant increases in the RNA ratios were obtained by X-irradiation at 14 mGy (air-kerma), corresponding to 18 to 19 mGy (EDw) of gamma-rays if the above-mentioned coefficient is used. In the case of *Cdkn1a* , the increased levels are not remarkable in humans [ 8 , 9 ] compared with mice [ 22 ]. For further estimation of low-dose irradiation, a more suitable RNA should be selected according to animal species.

Aminothiols, such as the radioprotector amifostine, neutralize reactive oxygen species generated by irradiation [ 40 ]. Pretreatment with cysteamine, an endogenous aminothiol, before X-irradiation decreased the *Bax* / *Myc* and *Bbc3* / *Myc* RNA ratios (Fig. 3 A, left, middle). The data are consistent with a previous report that DSB numbers in irradiated cells are decreased by pretreatment with another aminothiol [ 41 ]. The *Cdkn1a* / *Myc* RNA ratio was increased by cysteamine without irradiation (Fig. 3 A, right), suggesting that cysteamine directly induces growth arrest. For biodosimetry, apoptotic and growth arrest indicators should be separately quantified to predict DNA damage when using substances that affect cell growth.

### Studies using continuous irradiation models

The increase in the DDI gene/ *Myc* RNA ratios after a single irradiation (Fig. 4 A) was presumed to be delayed after continuous irradiation with the same total dose at a low dose rate. We studied continuous irradiation by keeping mice for 3 or 4 h in a field of 0.032 Gy (EDw)/h of gamma-rays (Fig. 4 C). Immediately after continuous irradiation for 4 h with a total dose of 0.128 Gy (EDw), the RNA ratios in WBCs reached almost the peak level, which was significantly different from the RNA ratios in the blood from non-irradiated mice. In the case of 3-h irradiation at a total dose of 0.098 Gy (EDw), peak RNA ratios were observed 1 to 3 h after the end of irradiation. All the peak levels were similar to those obtained 4 h after single irradiation of 0.1 Gy (Fig. 4 B). The results indicated that the peak levels depended on the total dose of EDW, either by continuous or single irradiation.

As another continuous irradiation model, ^32^ P-phosphate was injected into mice and the WBCs used for analysis of the DDI RNA ratios (Fig. 4 D). Injection at 200 and 80 MBq/kg corresponds to a dose rate of 0.080 and 0.032 Gy/h, respectively. When WBCs were collected 4 h after injection, the predicted total EDw was 0.320 Gy or 0.128 Gy. Similar to continuous irradiation with gamma-rays (Fig. 4 C), the RNA ratios were expected to approach the peak level obtained after single irradiation at the corresponding EDw. The 95% confidence interval (CI) of the RNA ratios predicted by the regression line (Fig. 4 B) obtained after single irradiation was calculated. The 95% CIs after 0.320 and 0.128 Gy irradiation are shown in Fig. 4 D (gray lines), and the obtained values of all the RNA ratios were within the range of the 95% CI. When WBCs were collected 8 h after injection, the *Bax* / *Myc* RNA ratio fell in the predicted ranges of 0.640 or 0.256 Gy. The *Bbc3* / *Myc* or *Cdkn1a* / *Myc* RNA ratios, however, were above or below the predicted range, respectively. The deviation from the predicted range may be due to the long 8-h continuous exposure in which subsequent biologic processes can be triggered by the expression of *Cdkn1a* , *Bax* and *Bbc3* mRNAs. These results demonstrated that the DDI RNA ratios in WBCs can be used to predict exposure doses after continuous irradiation. Further quantitative studies are needed, however, to determine more precise dosimetry.

### Future application

Quantitative sptudies of RNA ratios focusing on undifferentiated cells in circulating blood, as described above, could be used for several applications, including biodosimetric technology and molecular biologic studies, following various types of body irradiation. Because the activity of the animal does not affect the RNA ratios, this method can be applied to animals that are difficult to breed or handle, or to wild animals. Knowledge gained from this study could be applied to experiments using human specimens, and to blood isolated from patients in hospitals on bed rest or from first responders in disasters immediately after exposure.

## FUNDING

All the works in this paper were supported by National Institute of Radiological Sciences, funded by Ministry of Education, Culture, Sports, Science and Technology of Japanese Government. Funding to pay the Open Access publication charges for this article was provided by the National Institute of Radiological Sciences.

## Supplementary Material

Supplementary Data
