# Supplementary material for: Quantification of damage due to low-dose radiation exposure in mice: construction and application of a biodosimetric model using mRNA indicators in circulating white blood cells
Source: J Radiat Res. 2015 Nov 19;57(1):25–34. doi: 10.1093/jrr/rrv066 (PMC4708920; doi:10.1093/jrr/rrv066)
Supplement: Supplementary Data [file rrv066_Supplementary_Data.zip › rrv066supp.docx]

**Supplemental Figure 1**: (**A**) Scarcity of CD34-positive (precursor of lymphoid and myeloid, lower left panel) and CD117-positive (hematopoietic stem cell, lower right panel) undifferentiated cells in WBC (upper panels) by flow cytometry. Peripheral blood from mice during the daytime was treated with anti-mouse CD34 or CD117 rat monoclonal antibody (clones MEC14.7 or 2B8, respectively) conjugated with Alexafluor® 647 (AbD Serotonic, Kidlington, UK), lysed by VersaLyse^TM^ (Beckman Coulter, Inc.) and analyzed using FACSCalibur (Becton Dickinson). Identified cell numbers are written on the panels. (**B**) DDI gene/Gapdh RNA ratios before or after enrichment of undifferentiated WBC, as described below. Four hours after x-irradiation of 0 or 500 mGy (air-kerma, 4 mice/group), blood samples were collected and used (Before). After removal of RBC with ammonium chloride, WBC were incubated with Dynal® Mouse B cell-negative isolation kit (Invitrogen Dynal AS, Oslo, Norway) plus Dynal® Mouse T cell-negative isolation kit (Invitrogen Dynal AS) at 20℃ for 15 min to bind differentiated cells with beads. After magnetic removal of the differentiated cells at 4℃, the remaining cells as the enriched fraction of undifferentiated cells (After) were used for RNA analysis to quantitate the DDI gene/Gapdh RNA ratios. Means and standard errors among mice are shown. (**C**) Myc/Gapdh RNA ratios before or after enrichment of undifferentiated WBCs, as described above.

**Supplemental Figure 2**: The increase and decrease in the DDI gene/Myc RNA ratios in WBC following irradiation. Blood was collected from mice in quadruplicate at the indicated times after x-irradiation at an air-kerma dose of 0.514 Gy. Means and standard errors are shown.
