# Supplementary figures and images for: Quantification of damage due to low-dose radiation exposure in mice: construction and application of a biodosimetric model using mRNA indicators in circulating white blood cells
Source: J Radiat Res. 2015 Nov 19;57(1):25–34. doi: 10.1093/jrr/rrv066 (PMC4708920; doi:10.1093/jrr/rrv066)

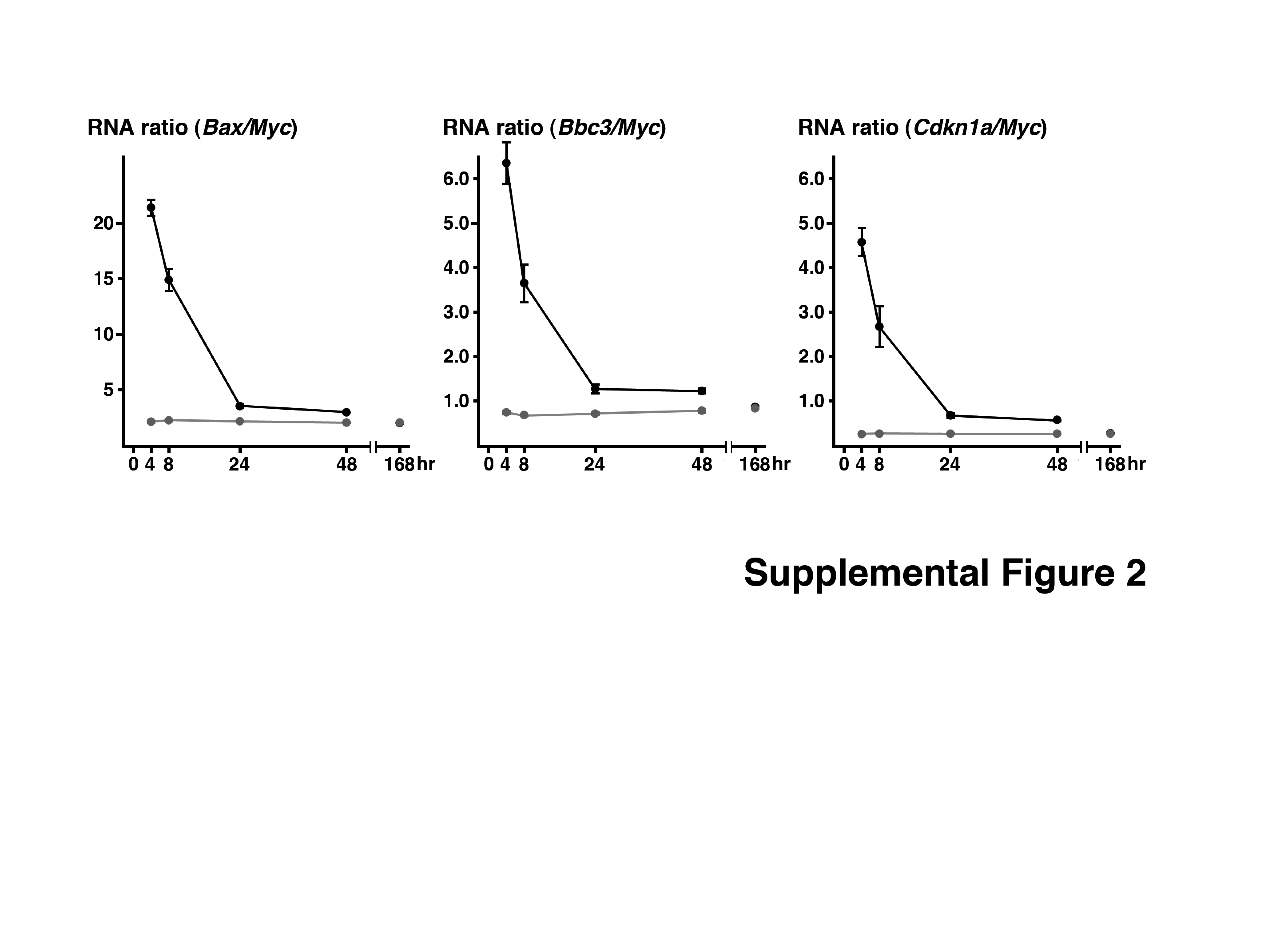

Supplement: Supplementary Data [file rrv066_Supplementary_Data.zip › rrv066supp_fig2.tif]

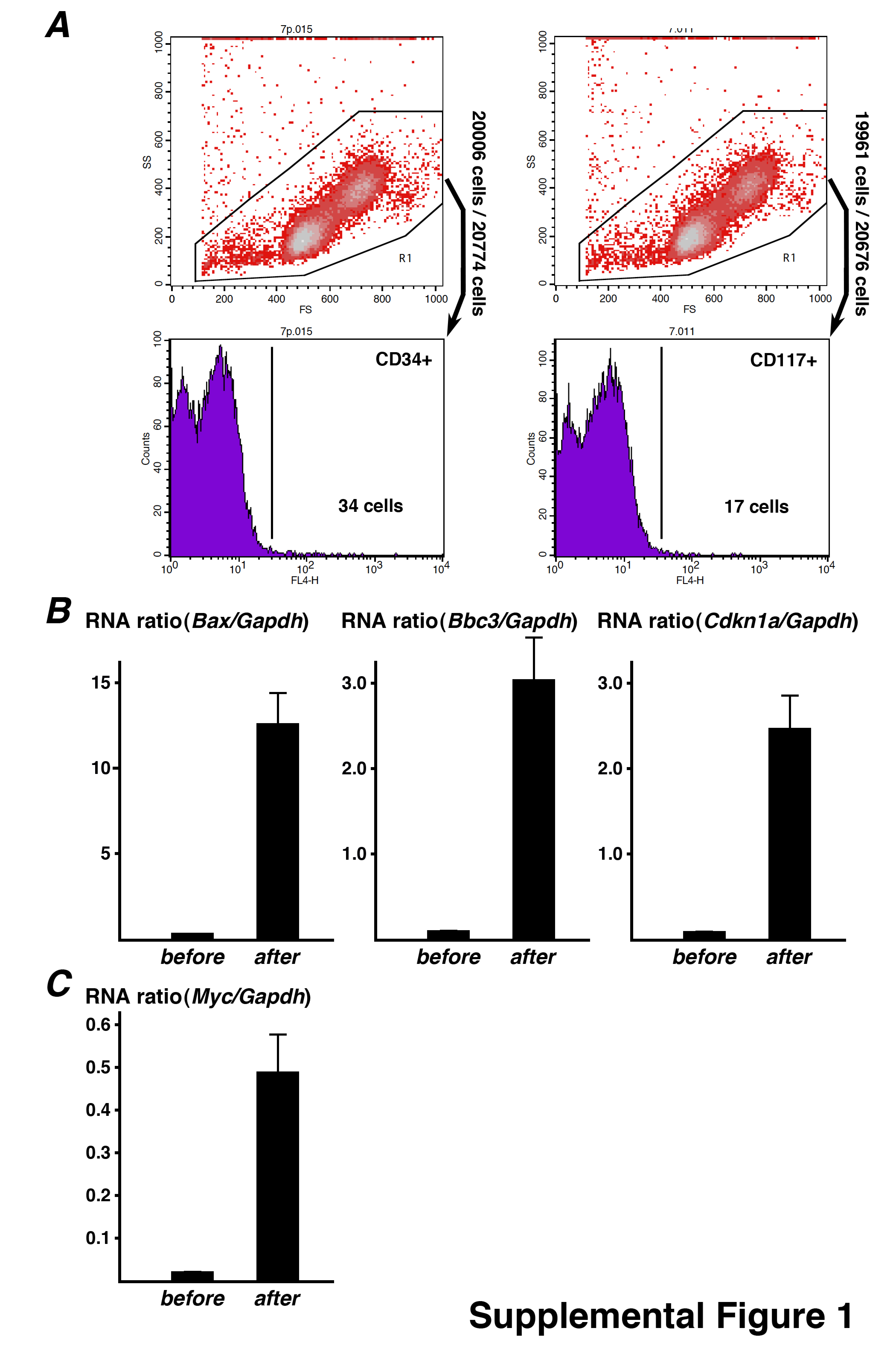

Supplement: Supplementary Data [file rrv066_Supplementary_Data.zip › rrv066supp_fig1.tif]
